# An Inquiry into the Pathological Importance of Ulceration of the Os Uteri

**Published:** 1855-04

**Authors:** 


For sale by MILLER, ORTON & MULLIGAN. H.

---

ART. VI.—*An Inquiry into the Pathological Importance of Ulceration of the Os Uteri. Being the Croonian Lectures for the year* 1854. By CHARLES WEST, M. D., F. R. C. P., etc. Philadelphia: Blanchard and Lea. 1854.

Very much such a book as the last under review (Gross on Foreign Bodies) is this thin octavo of 90 pages. Like it, it is statistical and full of facts, but unlike it, it will excite a deal of adverse criticism, and disturb the peace of many minds now possessed of quiet faith in the importance of those drusy, *framboiseé*, raspberry, and polypoid excrescences, so charmingly depicted by Prof. Meigs one year agone, as well as their reliance on the virtues of *nitras argenti*, whether applied by "indifferent, destructive, or antiphlogistic contact."

"Charles West, M. D.," is well known as the author of the most philosophical work on diseases of children yet published, and the high respect for his talent as an observer, which has resulted from the public appreciation of that work, will go far to sustain him in his present antagonistic position; for in these lectures he does assume a position of antagonism to all the recent teachings of Bennett, Simpson, and others who assume that nearly all uterine disorders, hypertrophy, induration, retroversion, anteversion, leucorrhœa menorrhagia, and the host of general symptoms depending upon them, are all attributable to inflammation of the os or cervix uteri, and to be cured by local applications thereto.

Dr. West's appearance in this discussion is that of a seeker after truth. For all that we can see he is innocent of any "axe to grind," neither does he so express himself as to imply any disrespect for the patient labors of those who have established the prevalent pathology. Conceding to them energy, talent, and sincerity, he still boldly asserts that the subject has never been carefully studied, and that all the statistics yet published are so deficient or unskillfully framed as to be useless for inductive reasoning.

Accordingly he resorts to personal research, and here is his

"Table showing the chief results of the examination of 62 uteri:

| | | |
|---|---|---|
| Uterus healthy in | | 33 |
| " diseased in | | 29 |
| Ulceration of the os uteri existed in | | 17 |
| " existed alone in | 11 | |
| " with diseased lining of the uterus in | 3 | |
| " with induration of the walls of the uterus in | 3 | — 17 |
| Induration of walls without ulceration of os, | | 5 |
| Disease of lining membrane of uterus without ulceration of os, | | 7 |
| Total of diseased uteri, | | 29 |

All of these uteri were taken from patients dying of other than uterine disease. The proportion of diseased organs in these circumstances is startling. It will be seen that 12 of 29 cases of disease had no ulceration, *ergo*, these did not depend upon inflammation of the os for their cause. Again, of 17 cases of ulceration 11 existed alone.

Another table is made up of living cases of patients having uterine symptoms. On examination it proved that of these, 125 had symptoms without ulceration, and 117 had symptoms with ulceration.

These are the main facts. Others like them are not wanting, and the entire investigations are conducted in a philosophical spirit which confers upon them great value. The question is again open, and the speculum will be more than ever in demand.

It strikes us that one thing has been neglected in these inquiries. Dr. West's statistics show that of 62 unsuspected women, 29 had uterine disease. We have known patients thus afflicted to leave their beds for the first time in months, go to water cures, and come home in a few weeks, to all appearance, well; while the *real condition of the uterus was not one whit improved, as demonstrated by examination.* Have not the confident assurances of help and the mental effect of local treatment a very salutary effect? Are not many of these cases of imaginary cures of trivial diseases, which under the strange sympathies of the sexual organs with the mind are magnified into most distressing maladies? This is our own conviction, for in no other way can we account for the fact that a woman of will and energy suffers but little in general health from ulceration of the uterus, while others are deprived of locomotion by identical conditions. The analogue to this fact is found in the comparative importance assigned by different patients to symptoms of spinal irritation.

